# Huntington's disease clinical trials update: August 2026

**DOI:** 10.1177/18796397261499360

**Published:** 2026-10-19

**Authors:** Mena Farag, Opeyemi Kinyomi, Sarah J Tabrizi, Edward J Wild

**Affiliations:** 1 Huntington's Disease Centre, 61554 UCL Queen Square Institute of Neurology, London, UK

**Keywords:** Huntington’s disease, clinical trials

## Abstract

In this edition, we highlight several important developments across the Huntington's disease therapeutic pipeline. We discuss the launch and subsequent discontinuation of POINT-HD, the first-in-human phase I study of RG6496 (Hoffmann-La Roche), an allele-selective antisense oligonucleotide designed to selectively lower mutant huntingtin while largely preserving wild-type huntingtin expression. We also discuss the launch of two phase III efficacy trials: INVEST-HD, evaluating votoplam (Novartis Pharmaceuticals), and PRECISE-HD, evaluating pridopidine (Prilenia). In addition, we summarise recent advances across the field, including preclinical updates for LQT-23 (LoQus23), SRP-1005 (Sarepta Therapeutics) and HRN001 (Harness Therapeutics); interim 12-month clinical results from SKY-0515 (Skyhawk Therapeutics); the announcement by uniQure that 3-year data from the phase I/II AMT-130 study may support a Biologics License Application seeking accelerated approval and Roche's discontinuation of the phase II GENERATION HD2 study of tominersen. Finally, we provide an updated listing of registered and ongoing clinical trials in Huntington's disease.

## Introduction

The Clinical Trials Update is a regular feature devoted to highlighting ongoing and recently completed clinical trials in Huntington's disease (HD). Clinical trials previously reviewed in this section are listed in Table 1. For current and completed studies discussed here, endorsement status by the European Huntington's Disease Network (EHDN) is also indicated.

**Table 1.** Clinical trials previously reviewed by the Huntington's disease clinical trials update.

	Trial name	Intervention	Endorsed by EHDN	Edition
NCT02519036	IONIS-HTTRx	IONIS-HTT_Rx_^a^	Yes	September 2017^1^
NCT02215616	LEGATO-HD	Laquinimod	Yes	
NCT02197130	Amaryllis	PF-02545920	Yes	
NCT02006472	PRIDE-HD	Pridopidine	Yes	
NCT03225833	PRECISION-HD1	WVE-120101	Yes	February 2018^2^
NCT03225846	PRECISION-HD2	WVE-120102	Yes	
NCT01795859	FIRST-HD	Deutetrabenazine	No	
NCT02481674	SIGNAL	VX15/2503	No	August 2018^3^
NCT00712426	CREST-E	Creatine	No	
NCT03761849	GENERATION-HD1	RG6042^a^	Yes	January 2019^4^
NCT03344601	PACE-HD	Physical activity	Yes	
NCT02535884	HD-DBS	Deep brain stimulation	Yes	June 2019^5^
NCT02453061	TRIHEP3	Triheptanoin	No	
NCT04120493	AMT-130	AAV5-miHTT	Yes	April 2020^6^
NCT04102579	KINECT-HD	Valbenazine	No	
NCT05111249	VIBRANT-HD	Branaplam	Yes	April 2022^7^
NCT04514367	ANX005	ANX-005	No	
NCT04406636	SHIELD HD	Observational study	Yes	
NCT03761849	GENERATION-HD1	Tominersen^a^	Yes	
NCT05032196	SELECT-HD	WVE-003	Yes	
NCT03225833	PRECISION-HD1	WVE-120101	Yes	
NCT03225846	PRECISION-HD2	WVE-120102	Yes	
NCT02481674	SIGNAL	Pepinemab^b^	No	November 2022^8^
NCT05358717	PIVOT-HD	PTC518	Yes	
NCT05686551	GENERATION HD2	Tominersen^a^	Yes	August 2023^9^
NCT05541627	AB-1001	AAVrh10.CAG.hCYP46A1^c^	No	
NCT05822908	VO659-CT01	VO659	No	March 2024^10^
NCT05111249	VIBRANT-HD	Branaplam	Yes	
NCT06254482	PIVOT-HD	PTC518 (extension study)	Yes	September 2024^11^
NCT06585449	ALN-HTT02	ALN-HTT02	Yes	March 2025^12^
NCT05107128 NCT05655520	DIMENSION PURVIEW	SAGE-718	Yes	
NCT06873334	FALCON-HD	SKY-0515	No	October 2025^13^
NCT06826612	N/A	SPK-10001	No	
*NCT07246941	**POINT-HD**	**RG6496**	**Yes**	**August 2026**
*NCT07326709	**INVEST-HD**	**Votoplam**	**Yes**	
*NCT07609108	**PRECISE-HD**	**Pridopidine**	**No**	

^a^ IONIS-HTTRx, RG6042 and tominersen refer to the same molecule.

^b^ VX15/2503 and pepinemab refer to the same molecule.

^c^ AAVrh10.CAG.hCYP46A1, BV-101 and AB-1001 refer to the same molecule.

^d^ Votoplam, HTT227 and PTC518 refer to the same molecule.

In this edition, we highlight several important developments across the Huntington's disease therapeutic pipeline. We discuss the launch and subsequent discontinuation of POINT-HD (NCT07246941),^14^ the first-in-human phase I study of RG6496 (Hoffmann-La Roche), an allele-selective antisense oligonucleotide (ASO) designed to selectively lower mutant huntingtin (mHTT) while largely preserving wild-type huntingtin expression. We also discuss the launch of two phase III efficacy trials: INVEST-HD (NCT07326709),^15^ evaluating votoplam (Novartis Pharmaceuticals), and PRECISE-HD (NCT07609108),^16^ evaluating pridopidine (Prilenia). In addition, we summarise recent advances across the field, including preclinical updates for LQT-23 (LoQus23),^17^ SRP-1005 (Sarepta Therapeutics)^18^ and HRN001 (Harness Therapeutics);^19^ interim 12-month clinical results from SKY-0515 (Skyhawk Therapeutics);^20^ the announcement by uniQure that 3-year data from the phase I/II AMT-130 study may support a Biologics License Application seeking accelerated approval from the US Food and Drug Administration (FDA),^21^ and Roche's discontinuation of the phase II GENERATION HD2 (NCT05686551)^22^ study of tominersen.^23^ Finally, we provide an updated listing of registered and ongoing clinical trials in Huntington's disease.

We tabulate all currently registered and ongoing clinical trials in Tables 2–4. For further details on the methodology used, please refer to the first edition of this series.^1^

**Table 2.** Pharmacological clinical trials registered at the World Health Organization (WHO) International Clinical Trials Registry Platform (ICTRP) for people with Huntington's disease (HD) since the first edition of the “Huntington's disease clinical trials corner”.

Registration ID	Trial name	Intervention	Mechanism of action	Population	Comparison	Main outcome	Study design	Estimated enrolment	Sponsor	Location(s)
NCT07609108*	PRECISE-HD	Pridopidine	Sigma-1 receptor activation	Manifest HD (not receiving antidopaminergic medications for ≥6 months before screening)	Placebo	Change from baseline to week 52 in composite UHDRS	Randomized, double-blind, placebo-controlled, parallel-group trial with 52-week treatment period followed by a 2-year open-label extension (phase III)	400	Prilenia (collaborator: Ferrer Internacional S.A.)	Sites not yet listed (multi-centre)
NCT07516899*	-	[14C] LPM3770164	VMAT2 inhibitor (radiolabelled compound)	Healthy male volunteers	None	Recovery of total radioactivity in urine and faeces; pharmacokinetics and metabolite identification of [14C] LPM3770164	Open-label, single-dose, single-group mass-balance (phase I)	8	Luye Pharma Group Ltd	China (single centre)
NCT07378644*	FALCON-HD	SKY-0515	mRNA splicing modulator designed to lower mHTT and PMS1	Early HD; UHDRS TFC ≥10, TMS ≥6, IS ≥70	Placebo	Change from baseline to week 72 in composite UHDRS z-score	Randomized, double-blind, placebo-controlled, dose-ranging, parallel-group (phase II/III)	400	Skyhawk Therapeutics, Inc.	Argentina, Brazil, Canada, Georgia (multi-centre)
NCT07344519*†	-	ER2001	Small interfering RNA	Adults with early manifest HD who completed study ER2001-001 (NCT06024265）	None	Incidence and severity of AEs and serious AEs	Open-label, dose-escalation, single-group (phase I)	8	ExoRNA Bioscience	China (single centre)
NCT07326709*	INVEST-HD	Votoplam	Small molecule splicing modulator	Early HD (CAG ≥40, UHDRS TFC=13, TMS 7-25, IS ≥90	Placebo	Change from baseline to month 36 in composite UHDRS	Randomized, double-blind, placebo-controlled, parallel-group (phase III)	770	Novartis Pharmaceuticals	Canada, Israel, South Korea, Taiwan, UK, USA (multi-centre)
NCT07246941*†	POINT-HD	RG6496	Intrathecally administered HTT-lowering therapy; SNP-targeted approach	HD gene-expansion carrierss with target SNP, CAG >39, UHDRS TFC ≥10, TMS ≥6, IS ≥70	Placebo	Safety and tolerability assessed by AEs, neurological assessment, C-SSRS and MoCA	Randomized, placebo-controlled, single-ascending-dose study with open-label extension (phase I)	40	Hoffmann-La Roche	Argentina, Australia, New Zealand (multi-centre)
NCT06873334	FALCON-HD	SKY-0515	mRNA splicing modulator designed to lower mHTT and PMS1	Early HD; UHDRS TFC ≥10, TMS ≥6, IS ≥70	Placebo	Changes in blood mHTT, brain volume (MRI) and UHDRS at 12 months	Randomized, double-blind, placebo-controlled, dose-ranging, parallel assignment	120	Skyhawk Therapeutics, Inc.	Australia, New Zealand (multi-centre)
NCT06853743	NAD-HD	Nicotinamide riboside	Vitamin B3 derivative; NAD^+^ precursor aiming to improve mitochondrial function, energy metabolism and neuroprotection	Early and moderate HD	Placebo	Change in composite UHDRS at 24 months	Randomized, double-blind, placebo-controlled, parallel assignment	120	Oslo University Hospital	Norway (single centre)
NCT06826612	-	Intraparenchymal infusion of SPK-10001 into caudate and putamen bilaterally	AAV-mediated gene therapy delivering therapeutic construct to striatum to modify disease progression	HD-ISS stage 1 or 2 HD; UHDRS TFC ≥11	Placebo/sham surgery	Change in UHDRS TFC; safety/TEAEs	Randomized, sequential dose-escalation, quadruple-masked, parallel assignment	53	Spark Therapeutics, Inc.	USA (multi-centre)
NCT06585449	-	ALN-HTT02	RNA interference selectively targeting exon 1 of *HTT* mRNA	HD-ISS stage 2 or early stage 3 HD	Placebo	Frequency of TEAEs in both the double-blind and open-label phases, monitored over a 12-month period	Randomized, double-blind, parallel assignment, single dose	54	Alnylam Pharmaceuticals	Canada, Germany, UK (multi-centre)
NCT05822908	VO659-CT01	Intrathecal administration of VO659	ASO targeting mutant *HTT* and ataxin RNA to reduce toxic protein levels	SCA1, SCA3, HD	None	Safety, tolerability, and pharmacokinetics of multiple doses	Open-label, non-randomized	68	Vico Therapeutics B.V.	Denmark, France, Germany, Israel, Netherlands, UK (multi-centre)
CTIS2024-518875-73-00	TEMET-HD	Metformin	Modulation of cellular energy metabolism via AMP-activated protein kinase activation to slow cognitive decline	HD	Placebo	Change in UHDRS cognitive score after 6 and 12 months	Randomized, double-blind, placebo-controlled	60	IIS La Fe	Spain(pending)
NCT06474650**†**	-	LPM3770164	VMAT2 inhibitor	Healthy controls	None	Pharmacokinetics pre-dose and up to 240 h post-dose	Randomized, open-label, two-period, double-crossover (phase I) study	16	Luye Pharma Group Ltd	China (single centre)
NCT06469853**†**	-	MBF-015	Histone deacetylase 1/2 inhibitor	Early and moderate HD	None	Safety and tolerability at 43 days	Open-label, single centre (phase IIa) study	10	Medibiofarma S.L.	Spain (single centre)
NCT06312189	-	Valbenazine	VMAT2 inhibitor	HD with chorea; participated in study NBI-98854-HD3006 (NCT04400331)	None	Number of participants with TEAEs up to week 106	Non-randomized, open-label	7	Neurocrine Biosciences	Canada (multi-centre)
NCT06254482	-	PTC518	Small molecule splicing modulator	Participants who completed the treatment period in PTC518-CNS-002-HD	None	Number of participants with TEAEs up to month 30; blood total HTT levels up to month 28	Randomized, double-blind, parallel assignment, extension (phase IIb) study	250	PTC Therapeutics	Australia, Austria, Canada, France, Germany, Italy, Netherlands, New Zealand, Spain, UK (multi-centre)
NCT06097780	-	Nestacell	Dental pulp stem cell	Early and moderate HD	Placebo	Efficacy at 1 year	Randomized, double-blind, parallel assignment, multiple dose	120	Azidus Brasil	N/S
NCT06024265**†**	-	ER2001	Small interfering RNA	Early HD	None	Safety at 6.5 months	Multiple dose, open-label trial	15	ExoRNA Bioscience	China (single centre)
2022-001565-12	-	PTC518	Small molecule splicing modulator	Premanifest, prodromal and early HD	None	Safety at 24 months, blood total HTT levels at 24 months	Randomized, double-blind, parallel assignment, multiple dose	250	PTC Therapeutics	France, Germany, Netherlands, UK, USA (multi-centre)
NCT05822908	-	VO659	CAG-targeting ASO	Early HD, mild-moderate SCA1, mild-moderate SCA3	None	Safety at 253 days	Open-label, non-randomized, sequential assignment, multiple ascending dose	65 (19 HD, 19 SCA1 and 27 SCA3)	VICO Therapeutics B.V.	France, Germany, Italy, Poland, the Netherlands, UK (multi-centre)
NCT04556656**†**	PROOF-HD	Pridopidine	Sigma-1 receptor activation	Early HD	Placebo	Change in function at 65 weeks	Randomized, double-blind, parallel assignment, single dose trial	499	Prilenia Therapeutics	Austria, Canada, Czechia, France, Germany, Italy, Netherlands, Poland, Spain, UK, USA (multi-centre)
NCT05686551**†**	GENERATION HD2	Tominersen	Non-allele-selective ASO	Prodromal and early HD	Placebo	Safety at 24 months	Randomized, double-blind, dose-finding trial	360	Hoffmann-La Roche	USA, Spain, more sites to be confirmed (multi-centre)
NCT05655520**†**	PURVIEW	SAGE-718	Positive allosteric modulator of NMDA	Premanifest, early and moderate HD	None	Safety at 13 months	Single-dose open-label trial	153	Sage Therapeutics	Canada, USA (multi-centre)
NCT03019289**†**	-	Pridopidine	Sigma-1 receptor activation	Healthy controls, early and moderate HD	None	Sigma-1 receptor occupancy	Multiple dose, open-label trial	23	Prilenia Therapeutics / Teva	Germany (single centre)
NCT02494778**†**	Open PRIDE HD	Pridopidine	Sigma-1 receptor activation	Early and moderate HD	Placebo	Efficacy at 106 weeks	Open-label extension	400	Prilenia Therapeutics / Teva	Australia, Austria, Canada, France, Germany, Italy, Netherlands, Poland, Russia, UK, USA (multi-centre)
NCT02006472**†**	PRIDE HD	Pridopidine	Sigma-1 receptor activation	Early and moderate HD	Placebo	Efficacy at 26 weeks	Randomized, double-blind, parallel assignment, dose-finding trial	408	Prilenia Therapeutics / Teva	Australia, Austria, Canada, Denmark, France, Germany, Italy, Poland, Russia, Netherlands, UK, USA (multi-centre)
NCT01306929**†**	OPEN-HART	Pridopidine	Sigma-1 receptor activation	HD	None	Safety up to 72 months	Randomized, placebo-controlled, dose-ranging, parallel-group study.	134	Prilenia Therapeutics / Teva	Canada, USA (multi-centre)
NCT05509153	NAC-preHD	NAC	Antioxidant	Premanifest HD	Placebo	Efficacy at 36 months	Randomized, double-blind trial	160	Western Sydney Local Health District	Australia (multi-centre)
ISRCTN56240656**†**	FELL-HD	Felodipine	Calcium channel blocker	Early HD	None	Safety at 62 weeks	Non-randomised, multiple dose trial	18	Cambridge University	UK (single centre)
NCT05358821**†**	SURVEYOR	SAGE-718	Positive allosteric modulator of NMDA	Early and moderate HD	Placebo	Change in cognition at 28 days	Double-blind, placebo-controlled, single dose design trial	69	Sage Therapeutics	USA (multi-centre)
NCT05358717**†**	PIVOT HD	PTC518	Small molecule splicing modulator	Premanifest, prodromal and early HD	Placebo	Safety at 113 days	Randomized, double-blind, placebo controlled, parallel assignment, multiple dose trial	162	PTC Therapeutics	France, Germany, Netherlands, UK, USA (multi-centre)
NCT05475483**†**	-	SOM-3355 (bevantolol hydrochloride)	Beta-blocker	Early and moderate HD	Placebo	Efficacy at 8 weeks	Randomized, double-blind, placebo-controlled, parallel assignment multiple-dose trial	129	SOM Biotech	France, Germany, Italy, Poland, Spain, Switzerland, UK (multi-centre)
ACTRN12621001755820	-	SLS-005 (trehalose)	Disaccharide	Early HD, ALS, SCA3	None	Efficacy at 24 weeks	Non-randomized, open-label	15-18 (4 ALS, 10 HD, 4 SCA3)	Seelos Therapeutics	Australia (two centres)
NCT05541627	-	AB-1001 (BV-101)	AAV encoding for CYP46A1, enzyme converting cholesterol to 24-OH-cholesterol	Early HD	None	Safety at week 52	Non-randomized, open-label, sequential, single ascending dose	18	AskBio/ BrainVectis	France (single centre)
NCT05107128**†**	DIMENSION	SAGE-718	Positive allosteric modulator of NMDA	Early and moderate HD	Placebo	Change in cognition at 84 days	Double-blind, placebo-controlled, single dose design	189	Sage Therapeutics	Australia, Canada, USA (multi-centre)
NCT05111249**†**	VIBRANT HD	Branaplam	Small molecule splicing modulator	Early HD	Placebo	Reduction of mHTT protein at week 17 Safety at 104 weeks	Double-blind, placebo-controlled multiple dose design	75	Novartis Pharmaceuticals	Belgium, Canada, France, Germany, Hungary, Italy, Spain, UK, USA (multi-centre)
NCT05032196**†**	SELECT-HD	WVE-003	Allele-selective ASO	Early HD	Placebo	Safety at 36 weeks	Randomized, double-blind, placebo-controlled, combined single ascending dose/multiple ascending dose trial	36	Wave Life Sciences Ltd	Australia, Canada, Denmark, France, Germany, Poland, Spain, UK (multi-centre)
NCT05243017	-	AMT-130	rAAV5-miHTT	Early HD	None	Safety at 6 months	Non-randomized, sequential ascending, multiple-dose trial	15	UniQure Biopharma B.V.	Germany, Poland, UK (multi-centre)
NCT04713982	-	Deutetrabenazine	VMAT2 inhibitor	HD with chorea	None	Change in speech outcome at 10 weeks	Single-arm open-label trial	30	Vanderbilt University Medical Center	USA (single centre)
NCT04826692	-	Metformin	Antihyperglycemic/ AMPK activator	Early and moderate HD	Placebo	Change in cognition at 52 weeks	Randomized, parallel assignment, double-blinded trial	60	Instituto de Investigación Sanitaria La Fe	Spain (single centre)
NCT04514367**†**	-	ANX005	C1q inhibitor	Early HD	None	Safety at 36 weeks	Single-dose open-label trial	28	Annexon, Inc	USA (multi-centre)
NCT04421339**†**	-	Melatonin	Melatonin receptor agonist	HD with sleep disturbance	Placebo	Sleep quality at 9 weeks	Randomised, cross-over, single-blinded (participant/caregiver)	20	The University of Texas Health Science Center, Houston	USA (single centre)
NCT04400331	-	Valbenazine	VMAT2 inhibitor	Early and moderate HD	None	Safety at 104 weeks	Open-label, single arm trial	150	Neurocrine Biosciences	USA, Canada (multi-centre)
NCT04301726	-	Deutetrabenazine	VMAT2 inhibitor	HD with dysphagia	Placebo	Dysphagia at 18 months	Randomized, parallel assignment, triple blinded trial	48	Fundación Huntington Puerto Rico	N/S
NCT04478734; CTIS2023-508637-14-00	HUNTIAM	Thiamine and biotin	B vitamins	HD	Moderate vs high doses of thiamine and biotin	Safety at 52 weeks	Randomized, parallel assignment, open-label trial	24	Fundación Pública Andaluza para la gestión de la Investigación en Sevilla	Spain (single centre)
NCT04201834**†**	-	Risperidone	Dopamine antagonist	Early and moderate HD with chorea	None	Change in motor scales at 12 weeks	Non-randomized, open-label (assessor-blind), uncontrolled trial	12	University of Rochester	USA (single centre)
NCT04071639	-	Haloperidol, risperidone, sertraline and coenzyme Q10	Multiple (dopamine antagonists, selective serotonin reuptake inhibitor, dietary supplement)	Early and moderate HD	Coenzyme Q10	Efficacy at 5 years	Randomized, open-label, controlled, parallel trial	100	Second Affiliated Hospital, School of Medicine, Zhejiang University	China (single centre)
NCT04120493	AMT-130	rAAV5-miHTT	Non-allele-selective miRNA	Early HD	Sham intervention	Safety at 18 months	Randomized, double-blind, sham-controlled, parallel trial	26	UniQure Biopharma B.V.	USA (multi-centre)
NCT04102579**†**	KINECT-HD	Valbenazine	VMAT2 inhibitor	HD with chorea	Placebo	Efficacy at 12 weeks	Randomized, double-blind, placebo-controlled, parallel trial	120	Neurocrine Biosciences, Huntington Study Group	USA (multi-centre)
EUCTR2019-002178-30-DK**†**	-	WVE-120102	Allele-selective ASO	HD	None	Safety and tolerability at 97 weeks	Open-label extension	70	Wave Life Sciences Ltd	Australia, Canada, Denmark, France, Poland, UK (multi-centre)
NCT04000594**†**	GEN-PEAK	RG6042	Allele-nonselective ASO	HD	None	Pharmacodynamics and pharmacokinetics at multiple timepoints until 6 months	Non-randomized. open-label, multiple-dose, parallel trial	20	Hoffmann-La Roche	Netherlands, UK (multi-centre)
NCT03980938**†**	-	Neflamapimod	p38α MAPK inhibitor	Early HD	Placebo	Change in cognitive scales at 10 weeks	Randomized, double-blind, placebo-controlled, cross-over trial	16	EIP Pharma Inc, Voisin Consulting, Inc.	UK (single centre)
NCT03842969**†**	GEN-EXTEND	RG6042	Allele-nonselective ASO	HD	None	Safety and tolerability at up to 5 years	Open-label extension	1050	Hoffmann-La Roche	USA, Canada, Europe (multi-centre)
NCT03761849**†**	GENERATION-HD1	RG6042	Allele-nonselective ASO	HD	Placebo	Clinical efficacy at 101 weeks	Randomized, double-blind, placebo-controlled, parallel trial	909	Hoffmann-La Roche	USA, Canada, Europe (multi-centre)
NCT03515213**†**	-	Fenofibrate	PPARα agonist	HD	Placebo	Pharmacodynamics at 6 months	Randomized, double-blind, placebo-controlled, parallel trial	20	University of California, Irvine	USA (single centre)
NCT03764215	Tasigna HD	Nilotinib	Selective Bcr-Abl tyrosine kinase inhibitor	HD	None	Safety, tolerability and pharmacodynamics at 3 months	Open-label, multiple ascending dose	20	Georgetown University	USA (single centre)
NCT03225833**†**	PRECISION-HD1	WVE-120101	Allele-selective ASO	HD	Placebo	Safety and tolerability at 1 and 120 days	Randomized, double-blind, placebo-controlled, combined single ascending dose/multiple ascending dose trial	48	Wave Life Sciences Ltd	Australia, Canada, Denmark, France, Poland, UK (multi-centre)
NCT03225846**†**	PRECISION-HD2	WVE-120102	Allele-selective ASO	HD	Placebo	Safety and tolerability at 1 and 120 days	Randomized, double-blind, placebo-controlled, combined single ascending dose/multiple ascending dose trial	60	Wave Life Sciences Ltd	Australia, Canada, Denmark, France, Poland, UK (multi-centre)
NCT02453061**†**	TRIHEP 3	Triheptanoin	Anaplerotic therapy	HD	Safflower oil	Pharmacodynamic efficacy at 6 months	Randomized, double-blind, controlled, parallel trial	100	Institut National de la Santé Et de la Recherche Médicale, Ultragenyx Pharmaceutical Inc	France, Netherlands (multi-centre)
NCT02509793	-	Tetrabenazine	VMAT2 inhibitor	HD with impulsivity	None	Cognitive and behavioural effects at 8 weeks	Single group, open-label trial	20	University of Texas Health Science Center, and H. Lundbeck A/S	USA (single centre)
NCT02481674**†**	SIGNAL	VX15/2503	Anti-semaphorin 4D monoclonal antibody	Late premanifest or early HD	Placebo	Safety and tolerability at 15 and 21 months	Randomized, double-blind, placebo-controlled, parallel trial	240	Vaccinex Inc., Huntington Study Group	USA (multi-centre)
EUCTR2013-002545-10-SE	OSU6162Open1309	(-)-OSU616	Monoaminergic stabilizer	HD, PD, brain trauma, stroke, myalgic encephalomyelitis and narcolepsy	None	Safety at 3, 6 and 12 months	Single group, open-label trial	240	A. Carlsson Research AB	Sweden (multi-centre)
NCT00514774	UDCA-HD	Ursodiol	Bile acid	HD	Placebo	Safety, tolerability, and pharmacokinetics at 35 days	Randomized, double-blind, placebo-controlled, parallel trial	21	Oregon Health and Science University, Huntington Study Group, Huntington Society of Canada	N/S

AAV: adeno-associated virus; AMP: adenosine monophosphate; AMPK: AMP-activated protein kinase; ASO: antisense oligonucleotide; C-SSRS: Columbia-Suicide Severity Rating Scale; HD: Huntington's disease; HD-ISS: Huntington's Disease Integrated Staging System; HTT: huntingtin; IS: Independence Score; MoCA: Montreal Cognitive Assessment; mRNA: messenger ribonucleic acid; NAC: N-acetylcysteine; NAD^+^: nicotinamide adenine dinucleotide (oxidised form); NMDA: N-methyl-D-aspartate; N/S: not specified; RNA: ribonucleic acid; PD: Parkinson's disease; PPARα: peroxisome proliferator-activated receptor alpha; SCA1: spinocerebellar ataxia 1; SCA3: spinocerebellar ataxia 3; SNP: single nucleotide polymorphism; TD: tardive dyskinesia; TEAEs: treatment-emergent adverse events; TFC: Total Functional Capacity; TMS: Total Motor Score; UHDRS: Unified Huntington's Disease Rating Scale; VMAT2: vesicular monoamine transporter 2. *Note:* IONIS-HTT_Rx_, ISIS 443139, RG6042 and tominersen refer to the same molecule. Votoplam, HTT227 and PTC518 refer to the same molecule. New trials added since the last Clinical Trials Update are indicated by *. Pharmacological trials terminated are indicated by †.

**Table 3.** Invasive non-pharmacological clinical trials registered at the WHO ICTRP for people with HD since the first edition of the “Huntington's disease clinical trials corner”.

Registration ID	Trial name	Intervention	Mechanism of action	Population	Comparison	Main outcome	Study design	Estimated enrolment	Sponsor	Location(s)
NCT07513844*	-	Low-Intensity Focused Ultrasound Stimulation (LIFUS) targeting the GPe	Non-invasive neuromodulation of basal ganglia circuitry via focused ultrasound; intended to modulate pallidal activity and reduce HD motor symptoms	HD-ISS stages 2 or 3	None	Safety (incidence of dose-limiting toxicity); efficacy assessed by change in UHDRS TMS	Non-randomized, sequential assignment, single-masked safety and feasibility trial (phase I/II)	24	First Affiliated Hospital of Fujian Medical University	China (single centre)
NCT07451613*	REGEN4HD	hNSC-01 (human neural stem cell transplantation into the striatum)	Cell replacement and neurorestorative therapy	Genetically confirmed HD	None	Safety and tolerability assessed by TEAEs	Open-label, non-randomized, sequential dose-escalation study (phase Ib/IIa)	21	Leslie Thompson, University of California, Irvine	USA (single centre)
NCT06444217	FibroTG-HD	Skin biopsy	Skin biopsy	Individuals with a CAG ≥36 allele (with reduced or full penetrance)	None	In vitro validation of a RNA trans-splicing gene therapy for the correction of supernumerary CAG repeats into fibroblasts derived from skin biopsies	Open-label, single group assignment	20	University Hospital, Angers	France (single centre)
NCT06097780	-	Nestacell	Dental pulp stem cell	Early and moderate HD	Placebo	Efficacy at 1 year	Randomized, double-blind, parallel assignment, multiple dose	120	Azidus Brasil	N/S
NCT04244513	-	GPi DBS	DBS	HD with chorea	Sham intervention	Efficacy at 3 and 6 months	Randomized, double-blind, sham-controlled, cross-over trial	40	Beijing Municipal Administration of Hospitals, Medtronic	China (multi-centre)
NCT04219241	ADORE-EXT	Cellavita	Stem cell therapy	HD	None	Efficacy and safety at 2 years	Open-label extension	35	Azidus Brasil, Cellavita Pesquisa Científica Ltda	Brazil (single centre)
ISRCTN52651778	TRIDENT	Foetal stem cell transplant	Stem cell therapy	Early HD	Usual care	Safety at 4 weeks	Randomized, open-label, controlled, parallel trial	30	Cardiff University, UK	UK (single centre)
NCT02728115	SAVE-DH	Cellavita	Stem cell therapy	HD	None	Safety at 5 years	Non-randomized, open-label, uncontrolled, parallel trial	6	Azidus Brasil	Brazil (single centre)
NCT03252535**†**	ADORE-HD	Cellavita	Stem cell therapy	HD	Placebo	Efficacy at 120 days	Randomized, double-blind, placebo-controlled, parallel trial	35	Azidus Brasil	Brazil (single centre)
NCT03297177	-	Autologous stem/stromal cells	Autologous stem/stromal cell injection	HD, AD, PD, CBD, MS	None	Safety at 5 years	Single group, open-label trial	300	Healeon Medical Inc, Global Alliance for Regenerative Medicine, Regeneris Medical	Honduras, USA (multi-centre)
NCT02535884	HD-DBS	GP DBS	DBS	Moderate HD with chorea	Sham intervention	Efficacy at 12 months	Randomized, double-blind, sham-controlled, parallel trial	50	Heinrich-Heine University, KKS Netzwerk, Medtronic, The George Institute, EHDN, CHDI Foundation, Inc.	Austria, France Germany, Switzerland (multi-centre)
NCT01834053	BMACHC	Bone marrow derived MNC transplant	Bone marrow transplant	HD with chorea	None	Cognitive and behavioural effects at 6 months	Single group, open-label trial	50	Chaitanya Hospital, Pune	India (single centre)
NCT02252380	-	MR-guided focused ultrasound	Extracranial stereotactic radioablation	HD, ET, HT, PD, WD, dystonia, TD, or orofacial dyskinesias	None	Adverse events after the procedure	Single group, open-label trial	10	InSightec	Canada (single centre)

AD: Alzheimer's disease; CBD: corticobasal degeneration; DBS: deep brain stimulation; ET: essential tremor; GP: globus pallidus; GPe: external globus pallidus; GPi: internal globus pallidus; HD: Huntington's disease; HT: Holmes tremor; ICTRP: International Clinical Trials Registry Platform; MNC: mononuclear cells; MR: magnetic resonance; MS: multiple sclerosis; N/S: not specified; RNA: ribonucleic acid; PD: Parkinson's disease; TD: tardive dyskinesia; TEAEs: treatment-emergent adverse events; TFC: Total Functional Capacity; TMS: Total Motor Score; UHDRS: Unified Huntington's Disease Rating Scale; WD: Wilson's disease. **Invasive non-pharmacological trials terminated are indicated by †.**

**Table 4.** Non-invasive non-pharmacological clinical trials registered at the WHO ICTRP for people with HD since the first edition of the “Huntington's disease clinical trials corner”.

Registration ID	Trial name	Intervention	Mechanism of action	Population	Comparison	Main outcome	Study design	Estimated enrolment	Sponsor	Location(s)
NCT07409597*	MAMOC-MH	Neuropsychological testing and eye-tracking assessment	Identification of oculomotor markers associated with autobiographical memory and episodic future thinking deficits in HD	Presymptomatic and stage 1-2 HD patients, and matched healthy controls	Healthy controls	Autobiographical memory and future thinking performance assessed using TEMPau specificity scores and eye-tracking measures	Open-label, non-randomized, parallel-group observational study	80	University Hospital, Angers	France (single centre)
NCT07315984*	BioDigit HD-02	Wearable sensors and digital health assessments	Digital phenotyping/remote monitoring of motor, speech and cognitive symptoms	Adults genetically diagnosed with HD	Healthy controls	Change in daily walking duration and sit-to-stand transitions over 12 months	Prospective observational cohort study	75	BioSensics	USA (multi-centre)
NCT07253038*	The Social-HD Study	Double Movie for the Assessment of Social Cognition-Multiple Choice (DMASC-MC) social cognition assessment	Assessment of social cognition using dynamic, ecologically valid measures of emotion recognition and theory of mind	Adults with early manifest HD (CAG ≥40)	Healthy controls	Difference in DMASC-MC total score between HD and controls at baseline	Observational, (case-control, cross-sectional)	40	Region Skåne	Sweden (single centre)
NCT07213648*	-	MindTrails-Movement CBM-I mobile app	Cognitive bias modification for interpretation (CBM-I) targeting maladaptive threat interpretation to reduce anxiety	Adults with HD or PD with anxiety symptoms (NeuroQoL-Anxiety ≥12)	Waitlist control app (surveys + support resources, no CBM-I training)	Change in NeuroQoL-Anxiety short form score from baseline to 6 weeks	Randomized, open-label, parallel-group interventional pilot trial	136	University of Virginia	USA (single centre)
NCT07136844	Acti-Adult	Syde^®^ wearable device; standardised functional tests; disease-specific clinical scales	Digital mobility and activity monitoring with magneto-inertial sensors to capture real-world gait and upper limb function	Ambulatory adults with neurological or metabolic movement disorders (including HD) and obesity	Parallel disease cohorts	95^th^ centile of stride velocity (SV95C) every 6 months for up to 2 years	Non-randomized, parallel assignment, open-label; diagnostic purpose	300	Centre Hospitalier Universitaire de Liège	Belgium (single centre)
NCT07010705	MEND-HD	ActiGraph LEAP (wearable device); Axivity AX6 (wearable device)	Digital health technologies to quantify gait, mobility and chorea (wearable sensor-based endpoints)	HD-ISS stage 2 or early stage 3	Healthy controls	Known-group validity and inter-method reliability of digital measures of stride time variability and total time in chorea	Observational (case-control, prospective)	100	University of Rochester	USA (single centre)
NCT06843252	-	Active tDCS	Non-invasive brain stimulation modulating cortical excitability and frontostriatal networks to improve cognition and behaviour	Early and moderate HD	Sham tDCS (crossover)	Study completion, acceptability, side effects; secondary: cognition (FAB), behaviour (PBA-S)	Randomized, double-blind, sham-controlled, crossover pilot trial	16	The University of Texas Health Science Center at San Antonio	USA (single centre)
NCT06807892	CARE-MH	Synergistic management (simultaneous cognitive-motor exergames); sequential management (separate cognitive then motor training)	Cognitive-motor training via exergames aiming to improve neuroplasticity, balance, motor and cognitive function	Early HD	Synergistic vs sequential management	Berg Balance Scale (BBS)	Randomized, parallel-group, open-label supportive care trial	40	University Hospital, Angers	France (single centre)
NCT06774443	HT-HD	Neuropsychological assessment (Hinting Task)	Assessment of social cognition deficits, specifically Theory of Mind	HD	Healthy controls	Sensitivity of the Hinting Task in detecting social cognition impairments in HD	Observational (case-control, cross-sectional)	52	University Medical Center Groningen	Netherlands (single centre)
NCT06693466	HD-P1	Experimental pain assessment (pain facilitation, facial expression, and conditioned pain modulation)	Assessment of pain processing mechanisms and sensory modulation in HD	HD	None	Feasibility of experimental pain assessment protocols in HD	Observational (cohort, cross-sectional)	20	Leiden University Medical Center	Netherlands (single centre)
NCT06634628	iMagemHTT-009	PET imaging with novel radioligand [11C]CHDI-00491009	Radioligand binding to aggregated mHTT for quantification	HD	None	VT of [11C]CHDI-00491009 measured via PET imaging	Non-randomized, sequential assignment	27	CHDI Foundation, Inc.	Belgium (single centre)
NCT06626308	BIO-MH	Brain fMRI and gait assessment using 3D virtual reality	fMRI to assess early subcortical biomarkers and virtual reality-based gait analysis for detecting preclinical motor abnormalities	Premanifest HD	Healthy controls	BOLD signal changes in subcortical regions and alterations in gait parameters	Observational (case-control)	20	University Hospital, Grenoble	France (single centre)
NCT06626412	-	PET of SV2A using the radioligand 18F-SynVesT-1	Radioligand binding to SV2A for quantification of synaptic density	Late premanifest HD	Volumetric MRI	Baseline differences and longitudinal changes in synaptic density and brain volume	Non-randomized, single-group assignment	35	Universitaire Ziekenhuizen KU Leuven	Belgium (single centre)
NCT06585332**†**	-	Hand grip strength as a screening tool for respiratory dysfunction	Assessment of neuromuscular function related to respiratory effort	HD	None	Maximal hand grip strength, voluntary peak cough flow, maximal expiratory pressure, maximal inspiratory pressure	Observational (case only)	70	General University Hospital	Prague (single centre)
NCT06546488	CAT-HD	Behavioral assessments	Standardised assessment batteries using the DSCT and SAGE	HD	SDMT, SWR	SDMT compared to DSCT; SAGE score compared to SDMT and SWR scores	Observational (case only)	76	Ohio State University	USA (single centre)
NCT06490367**†**	TREHD	TRE diet	TRE diet, specifically maintaining a 6-8-h eating window every day for 12 weeks	Premanifest, prodromal and early HD	None	Change from baseline in the daily eating period at week 13	Prospective interventional, open-label, single-arm trial	25	Oregon Health and Science University	USA (single centre)
NCT06414967	MUSIC-HD	Music therapy	Music therapy using a digital music therapy tool combined with conventional management	Early HD	None	Change in irritability after 3 months	Single group, open-label	15	Poitiers University Hospital	France (single centre)
RBR-75ys4s9	-	Dance therapy	Dance sessions offered by physiotherapists using auditory and visual stimuli	HD, PD, dystonia	Standard medical treatment	Quality of life improvement	Two-arm randomized controlled trial with a blinded examiner	100	Universidade Federal de Juiz de Fora	Brazil (single centre)
ChiCTR2300069844	-	Repetitive transcranial magnetic stimulation	Transcranial magnetic stimulation	HD	None	EEG	Non-randomized, open-label, single group trial	20	Shenzhen People's Hospital	China (single centre)
ISRCTN47330596	-	Psychological intervention	Guided self help	Premanifest and manifest HD	Usual treatment	Feasibility at 3 and 6 months	Interventional randomized controlled trial	30	Leicestershire Partnership NHS Trust, UK	UK (single centre)
RBR-463yhb3	-	Multimodal physiotherapy	Balance intervention with rhythmic cues	HD	Educational program	Balance	Randomized, double-blinded, parallel assignment trial	36	São Paulo University, Brazil	Brazil (single centre)
ACTRN12622000908730	-	Online platform	Computerized cognitive training	Premanifest and early HD	Lifestyle education	Change in cognition at 12 weeks	Randomized, blinded (investigator, statistician) parallel assignment trial	50	Monash University, Australia	Australia (two centres)
ISRCTN11906973	HD-DRUM	Training app	Drumming	Premanifest, early and moderate HD	Standard medical care	Feasibility	Randomized, parallel assignment trial	50	Cardiff University, UK	UK (single centre)
NCT05326451	-	Transcranial direct current stimulation	Transcranial electrical stimulation	Early and moderate HD	None	Treatment completion, acceptability and safety	Non-randomized, open-label, single group trial	10	The University of Texas Health Science Center, Houston, USA	USA (single centre)
ACTRN12622000345785	-	Multidisciplinary therapy coaching program	Education	Premanifest and early HD	Lifestyle guidance	Barriers and motivators to engagement in telehealth interventions and digital health literacy	Randomized, single blind, parallel assignment trial	84	Perpetual limited	Australia (two centres)
NCT04917133	HUNT'ACTIV	Adapted physical workshops plus classic 4-week rehabilitation program	Physical activity, cycling, horse riding, situation tests, cultural outings	Mid-stage HD	Classic 4-week rehabilitation program	Motor function at 1 month	Randomized, parallel assignment trial	32	Assistance Publique - Hôpitaux de Paris	France (single centre)
NCT04429230	-	Transcranial pulsed current stimulation	Transcranial electrical stimulation	HD	Sham intervention	Feasibility at one year	Randomized, crossover double-blinded trial	15	Western University, Canada	N/S
ACTRN12620000281998	-	Ketogenic diet	Ketogenic diet	HD	None	Change in cognition and motor scores at 12 weeks	Non-randomized, open-label, single group trial	10	Waikato Hospital	New Zealand (-)
ACTRN12619000870156	-	Transcranial alternating current stimulation	Transcranial magnetic stimulation	Premanifest and early HD	Sham intervention	Biomarkers	Randomized, open-label, cross-over trials	60	Monash University, Epworth Centre for Innovation in Mental Health	Australia (single centre)
ACTRN12618001717246	-	Multidisciplinary therapy program	Exercise, cognitive training, lifestyle guidance and social activities	Premanifest HD	Standard of care	Feasibility and safety	Clustered, non-randomized, open-label, parallel trial	40	Edith Cowan University, Deakin University and Lotterywest	Australia (two centres)
NCT03417583	-	Neuropsychiatric treatment protocol	Multidisciplinary intervention	HD with neuropsychiatric symptoms	Standard of care	Change in quality of life at 18 months	Non-randomized, assessor-blinded, parallel trial	100	Vanderbilt University Medical Center and Teva Pharmaceuticals USA	USA (single centre)
CTRI/2018/01/011359	-	Repetitive transcranial magnetic stimulation	Transcranial magnetic stimulation	Early to moderate HD and PD	Sham stimulation	Efficacy at 5 days	Randomized, single-blind, placebo-controlled, parallel trial	40	Vinay Goyal	India (single centre)
NCT03344601	PACE-HD	Supported structured aerobic exercise training program	Physiotherapy	HD	Activity as usual	Data completeness, recruitment, retention, safety, adherence, fidelity and acceptability at 12 months	Nested open-label, randomized controlled parallel trial	120	Cardiff University and CHDI Foundation, Inc	Germany, Spain, USA (multi-centre)
ACTRN12617001269325	-	Swallowing skill training	Speech and language therapy	HD and ALS	None	Swallowing function and quality of life at 2 weeks	Single group, open-label trial	54	University of Canterbury	New Zealand (single centre)

3D: three-dimensional; AD: Alzheimer's disease; ALS: amyotrophic lateral sclerosis; BOLD: blood oxygen level-dependent; DSCT: Digit Symbol Coding Test; EEG: electroencephalography; ET: essential tremor; FAB: Frontal Assessment Battery; fMRI: functional magnetic resonance imaging; HD: Huntington's disease; HT: Holmes tremor; ICTRP: International Clinical Trials Registry Platform; mHTT: mutant huntingtin; MRI: magnetic resonance imaging; MS: multiple sclerosis; N/S: not specified; PBA-S: Problem Behaviors Assessment - Scale; PD: Parkinson's disease; PET: positron emission tomography; SAGE: Self-Administered Gerocognitive Examination; SDMT: Symbol Digit Modalities Test; SV2A: synaptic vesicle glycoprotein 2A; SWR: Stroop Word Reading; TD: tardive dyskinesia; tDCS: transcranial direct current stimulation; TRE: time-restricted eating; VT: volume of distribution; WHO: World Health Organization. New trials since the last Clinical Trials Update are indicated by *. Non-invasive non-pharmacological trials terminated are indicated by †.

If you would like to draw attention to specific trials, please feel free to e-mail us at: mena.farag@ucl.ac.uk and e.wild@ucl.ac.uk.

## Ongoing clinical trials

A list of all registered clinical trials is given in Tables 2, 3 and 4.

**RG6496 (NCT07246941)** ^14^

**Study Title:** A Study to Evaluate the Safety, Tolerability, Pharmacokinetics, and Pharmacodynamics of RG6496 in Huntington's Disease (POINT-HD).

**Intervention:** RG6496 is a selective ASO designed to lower mHTT protein while largely preserving expression of the wild-type protein. It is administered intrathecally and achieves allele selectivity by targeting a single nucleotide polymorphism (SNP) present on the expanded huntingtin gene (*HTT*) allele. This first-in-human, phase I interventional study intended to evaluate the safety, tolerability, pharmacokinetics and pharmacodynamics of single ascending doses of intrathecally administered RG6496 in HD gene-expanded carriers with confirmed target SNP status. The study comprised two parts: an initial randomised, placebo-controlled, double-blind, single-ascending dose phase, followed by an open-label extension for participants who complete the first part. The estimated enrolment was 40 participants. Primary outcome measures were safety and tolerability, including adverse events (AEs), changes from baseline in neurological examination, suicidal ideation or behaviour, and cognitive performance as measured by the Montreal Cognitive Assessment (MoCA). Secondary outcome measures included plasma concentrations of RG6496, the presence of anti-RG6496 antibodies in plasma and change from baseline in cerebrospinal fluid (CSF) mHTT concentrations.

**Sponsor/Funders:** Hoffmann-La Roche

**EHDN Endorsed:** Yes

**Comments:** Unlike non-selective HTT-lowering approaches, RG6496 was designed to selectively target the mutant HTT transcript while sparing expression of wild-type huntingtin. On the basis of large-scale genetic datasets, including Roche's global HD epidemiology programme (NCT06667414),^24^ a community letter released by Roche on 15^th^ December 2025 reported that approximately 40% of people with HD carry this target SNP on their expanded *HTT* allele;^25^ and only these individuals were eligible for participation in this clinical trial of RG6496 (POINT-HD).^14^

On 9^th^ July 2026, Roche announced the discontinuation of the phase I POINT-HD study.^23^ POINT-HD was terminated following new findings from a longer-term non-clinical study indicating that RG6496 could not be administered chronically with repeated dosing. Roche stated that there were no safety concerns associated with administration of a single dose in humans, but elected to discontinue the study because long-term treatment could no longer be considered. At the time of discontinuation, three participants had been enrolled and will continue protocol-defined follow-up. Analysis of the biomarker and clinical data generated from these participants may still provide valuable information to inform the future development of allele-selective therapeutic strategies for HD.

**Votoplam (NCT07326709)** ^15^

**Study Title:** A Study to Investigate the Efficacy, Safety and Tolerability of Votoplam in Participants With Huntington's Disease (INVEST-HD).

**Intervention:** Votoplam, also referred to as HTT227 (and formerly known as PTC518), is an orally administered small-molecule HTT-lowering therapy now being developed by Novartis for HD. INVEST-HD is a randomised, placebo-controlled, double-blind, phase III interventional study designed to evaluate whether votoplam slows disease progression in participants with early symptomatic HD compared with placebo. Participants are randomised in a 3:2 ratio to oral votoplam or placebo, with a variable double-blind treatment duration of up to 36 months, followed by a safety follow-up visit for participants not entering a separate open-label extension. The estimated enrolment is 770 participants. The primary outcome measure is change from baseline in composite Unified Huntington's Disease Rating Scale (cUHDRS) score at month 36. Secondary outcome measures include change from baseline in UHDRS Total Functional Capacity (TFC), Independence Scale (IS), Total Motor Score (TMS), Symbol Digit Modalities Test (SDMT) and Stroop Word Reading (SWR) test scores, time to functional decline, percent change from baseline in blood mHTT protein, change from baseline in serum neurofilament light chain (NfL) and the incidence and severity of treatment-emergent and serious AEs leading to participant withdrawal from the study.

**Sponsor/Funders:** Novartis Pharmaceuticals

**EHDN Endorsed:** Yes

**Comments:** Initiation of the phase III INVEST-HD study (NCT07326709)^15^ follows encouraging results from the phase II programme (NCT05358717)^26^ and its long-term extension (NCT06254482).^27^ On 28^th^ April 2026, PTC Therapeutics reported interim 24-month analysis of the long-term extension to the earlier PIVOT-HD study, suggesting dose-dependent slowing of clinical progression for HD-ISS stage 2 participants, with reported reductions in cUHDRS decline of 52% and 28% in the 10 mg and 5 mg dose groups, respectively, compared with an ENROLL-HD propensity-weighted natural-history comparator cohort.^28^ These findings were accompanied by sustained HTT-lowering, no treatment-related increases in NfL concentrations and a favourable safety profile. Potential signals of slowed progression were also observed in HD-ISS stage 3 participants. Novartis has subsequently initiated the global phase III INVEST-HD study, which will help determine whether the dose-dependent biomarker and clinical signals observed in the phase II programme can be confirmed in a large placebo-controlled trial and whether sustained HTT-lowering translates into clinically meaningful slowing of disease progression in early symptomatic HD.

**Pridopidine (NCT07609108)** ^16^

**Study Title:** Pridopidine Phase 3 Study in Huntington's Disease (PRECISE-HD).

**Intervention:** Pridopidine, a selective sigma-1 receptor agonist, is an orally administered investigational therapy being evaluated for its potential to slow clinical decline in HD. PRECISE-HD is a randomised, placebo-controlled, double-blind, phase III interventional study evaluating pridopidine 45 mg twice daily in adults with HD who have not used anti-dopaminergic medications for at least six months before screening. The study comprises a one-year placebo-controlled period, during which participants receive pridopidine or placebo in a 1:1 ratio, followed by a two-year open-label extension in which eligible participants receive pridopidine. Treatment begins with a two-week once-daily titration period, followed by twice-daily dosing. The estimated enrolment is 400 participants. The primary outcome measure is change from baseline to week 52 in cUHDRS score. Secondary outcome measures include change from baseline to week 52 in TFC, quantitative motor finger-tapping inter-onset interval and SWR score. Safety is assessed throughout the placebo-controlled and open-label extension periods.

**Sponsor/Funders:** Prilenia; Ferrer Internacional S.A.

**EHDN Endorsed:** No

**Comments:** PRECISE-HD seeks to build on findings from numerous large trials of pridopidine in HD that failed to demonstrate efficacy, most recently the phase III PROOF-HD study (NCT04556656),^29^ which evaluated pridopidine in early manifest HD. Although the primary and key secondary endpoints (change in TFC and cUHDRS at week 65) were not met in the overall study population, post-hoc and sensitivity analyses suggested potential benefit among participants who were not receiving antidopaminergic medications.^30^ PRECISE-HD (NCT07609108)^16^ has therefore been designed to prospectively evaluate pridopidine in a population restricted to individuals not treated with these medications. This study seeks to determine whether the signals observed in PROOF-HD can be confirmed in a dedicated phase III interventional study and further clarify the therapeutic potential of sigma-1 receptor agonism in HD.

## Breaking news

In this section we provide brief updates about ongoing or recently terminated clinical trials.

On 6^th^ January 2026, LoQus23 Therapeutics announced the nomination of LQT-23 as its lead development candidate for HD.^17^ LQT-23 is an orally administered, allosteric small-molecule inhibitor of MSH3/MutSβ, a DNA mismatch-repair protein complex implicated in somatic CAG repeat expansion,^31–34^ a key modifier of disease onset and progression in HD. According to the company, preclinical studies demonstrated potent and selective inhibition of MSH3/MutSβ activity and robust suppression of somatic expansion in cellular and animal models of HD. LQT-23 is currently in preclinical development, with LoQus23 indicating that regulatory filings are anticipated later this year.^17^

On 4^th^ February 2026, Sarepta Therapeutics announced regulatory approval from the New Zealand Medicines and Medical Devices Safety Authority (Medsafe) to initiate INSIGHTT, the first-in-human phase I study of SRP-1005 (formerly ARO-HTT), an investigational small interfering RNA (siRNA) therapy for HD.^18^ SRP-1005 is designed to reduce *HTT* expression following subcutaneous administration through a transferrin receptor 1 -mediated delivery platform intended to facilitate central nervous system (CNS) penetration. The phase I INSIGHTT study will evaluate the safety, tolerability and dose escalation of SRP-1005 in approximately 24 participants.^18^ The programme is noteworthy for its systemic subcutaneous delivery approach, which, if successful, could provide an alternative to intrathecal or neurosurgical administration strategies currently being investigated for HTT-lowering therapies. The trial will be important in determining whether the CNS distribution and HTT-lowering effects observed in preclinical studies can be translated into humans.

On 16^th^ February 2026, Harness Therapeutics also announced the nomination of HRN001 as its lead clinical candidate for HD and the establishment of a clinical advisory board to support progression towards first-in-human studies planned for 2027.^19^ HRN001 is a first-in-class ASO designed to increase expression of FAN1, a DNA repair nuclease that has emerged as a key genetic modifier of HD onset and progression.^31,35,36^ Unlike HTT-lowering strategies, HRN001 targets somatic CAG repeat expansion, a process well-recognised as a key driver of HD pathogenesis and progression.^36–40^ By upregulating FAN1 activity through a microRNA site-blocking antisense approach, the programme aims to stabilise the expanded HTT repeat and thereby modify this upstream driver of disease biology. As somatic expansion becomes an important therapeutic target in HD, clinical evaluation of HRN001 will provide an opportunity to test whether modulation of FAN1 can alter disease progression in people with HD.

As previously featured in the October 2025 update,^13^ Skyhawk Therapeutics has reported new interim data from the ongoing phase I/II study of SKY-0515. In a company press release issued on 1^st^ June 2026,^20^ twelve-month data showed dose-dependent reductions in blood mHTT protein of up to 69% and reductions in PMS1 mRNA of up to 26%. Treated participants demonstrated positive mean changes from baseline in cUHDRS scores over 12 months, compared with an expected decline derived from propensity-weighted analyses of Enroll-HD and TRACK-HD natural-history datasets. SKY-0515 was reported to be generally safe and well tolerated across dose levels studied. Skyhawk also announced completion of enrolment in the Australia and New Zealand component of the phase II/III FALCON-HD programme (004-ANZ), with 144 participants enrolled ahead of schedule, and expansion of the global FALCON-HD study (NCT07378644)^41^ to eight countries. Confirmation of these findings in the ongoing FALCON-HD programme will be important in determining whether the encouraging biomarker and clinical signals reported translate into clinically meaningful benefit.

On 17^th^ June 2026, uniQure announced that, following a Type B meeting with the FDA, the agency had indicated that the three-year analysis from the ongoing phase I/II AMT-130 study (NCT05243017)^42^ could serve as the primary basis for a Biologics License Application (BLA) seeking accelerated approval.^21^ The FDA also requested alignment on the design of a post-approval confirmatory study before submission of the application, including consideration of a concurrent standard-of-care control rather than a sham-surgery comparator.^21^ uniQure has stated its intention to submit a BLA in the third quarter of 2026. On 30^th^ April 2026, uniQure also announced that, following a pre-submission meeting with the UK Medicines and Healthcare products Regulatory Agency, they are intending to submit a Marketing Authorisation Application (MAA) for AMT-130 in the same quarter.^43^ According to the company, discussions focused on the clinical data package and manufacturing requirements to support the application and that the MAA would be based on three-year data from the US and European phase I/II studies, which reportedly demonstrated an approximately 75% slowing of disease progression at the higher dose, measured by the cUHDRS compared with a propensity score-matched external control.^43^ If these applications are accepted for review, AMT-130 would become the first gene therapy for HD to reach the regulatory submission stage, an important milestone in the development of disease-modifying therapies for HD.

On 9^th^ July 2026, Roche announced the discontinuation of the GENERATION HD2 (NCT05686551)^22^ study of tominersen in people with prodromal and early Huntington's disease.^23^ GENERATION HD2 was initiated following exploratory analyses of the phase III GENERATION HD1 study, which suggested that younger participants with lower disease burden might derive benefit from lower-dose, less frequent administration of tominersen.^44^ After completion of the planned 16-month treatment period in all 301 participants, top-line analyses showed that tominersen was well tolerated and achieved significant reductions in mHTT protein and NfL concentrations compared with placebo. However, these biomarker effects did not translate into a meaningful improvement in clinical outcomes, and Roche therefore discontinued further clinical development of tominersen.^23^ Although disappointing for participants and the HD community, these findings still provide important insights for the field. GENERATION HD2 confirms that sustained lowering of mHTT in humans, even if it lowers wild-type as well, and does not target *HTT* exon 1, is associated with robust NfL lowering - often taken as indicating reduced rates of neuroaxonal damage - but that clinical benefits, if they are to emerge, may take longer than 16 months. The study therefore refines, rather than resolves, key questions surrounding the therapeutic window, magnitude, duration, molecular target and anatomical distribution of HTT-lowering required to modify disease progression. Further analyses of the complete dataset, in which some participants were followed for up to 30 months, will help to inform the design of next-generation HTT-lowering approaches and the interpretation of future clinical trials.

## Data Availability

Data sharing not applicable as no datasets generated and/or analysed for this study.
